# Health-Related Fitness Levels among Title I Elementary School Students

**DOI:** 10.3390/ijerph18157778

**Published:** 2021-07-22

**Authors:** Taemin Ha, Jongho Moon, Brian Dauenhauer, Jennifer Krause, Jaimie McMullen, Karen Gaudreault

**Affiliations:** 1 School of Sport and Exercise Science, University of Northern Colorado, Greeley, CO 80639, USA; brian.dauenhauer@unco.edu (B.D.); jennifer.krause@unco.edu (J.K.); jaimie.mcmullen@unco.edu (J.M.); 2 Department of Physical Education, University of South Carolina, Columbia, SC 29208, USA; 3 Department of Health, Exercise and Sports Sciences, University of New Mexico, Albuquerque, NM 87131, USA; kgaudreault@unm.edu

**Keywords:** FitnessGram, physical education, assessment, low-income, children

## Abstract

Over the past few decades, studies have emphasized improving children’s health by increasing health-related fitness levels. Despite the known benefits of health-related fitness in youth, studies have also highlighted a lack of physical activity opportunities for children living in low-income households. The purpose of this study was to investigate the health-related fitness levels of students attending Title I (low-income) elementary schools. A total of 77 elementary students (50.6% female; *M*_age_ = 10.0, *SD* = 0.827) from two Title I elementary schools in the western United States completed the FitnessGram assessments of aerobic capacity, muscular strength and endurance, and flexibility. Descriptive statistics were used to identify the number of students in the Healthy Fitness Zone (HFZ) based upon the FitnessGram HFZ Performance Standards. Less than 17% of students achieved the HFZ for aerobic capacity and only 31.2% achieved the HFZ for upper body strength and endurance. Students performed better for abdominal strength and endurance and flexibility with 55.8% and 68.8% achieving the HFZ, respectively. The results of this study offer insights into the health-related fitness levels of a unique population, students attending Title I schools. School health professionals, including physical education teachers, need to be aware of existing disparities and make efforts to systematically intervene.

## 1. Introduction

Over the past few decades, many studies have emphasized improving children’s health through increasing health-related fitness levels [1,2,3]. Health-related fitness consists of five components: cardiorespiratory endurance, muscular strength, muscular endurance, body composition, and flexibility [4]. There are numerous benefits to optimal health-related fitness in elementary school-age children, including decreases in early-onset risk factors for cardiometabolic disease and a protective effect on obesity [5,6]. Additionally, physical fitness is associated with improvements in academic performance among children regardless of sociodemographic variables [7,8]. Thus, children’s health-related physical fitness is an influential factor in preventing multiple diseases and enhancing cognitive health.

World Health Organization 2020 Guidelines on Physical Activity and Sedentary Behaviour [9] and the latest edition of the Physical Activity Guidelines for School-Aged Children and Adolescents [10] include a recommendation that children accumulate at least 60 min of moderate to vigorous physical activity every day to keep their bodies healthy and maintain or improve current fitness levels. However, only 24% of children in the United States achieve this national guideline of physical activity [11]. A systematic review by Poitras et al. [12] reported that by the time students reached elementary school age, most were in poor cardiovascular shape due to a lack of physical activity. Even with clear indications that children attained health benefits from physical activity, the average health-related fitness levels in the United States have been declining as a result of various reasons, including inaction during leisure time, increased screen-based sedentary behavior, and widespread use of the automobile [13,14,15,16].

In the United States, in order to support students experiencing economic disadvantage, there is a federally funded program called Title I of the Elementary and Secondary Education Act (ESEA) of 1965. This program provides financial assistance to schools with high percentages of students from low-income families to assist in meeting the state academic standards [17]. Title I school status is determined by the percentage of students enrolled in free and reduced lunch programs and funds can be used to improve curriculum, instruction, and other school programs. According to the National Center for Education Statistics [18], approximately 21% of 5- to 17-year-olds were considered to fall under Title I status. Unfortunately, children from lower socioeconomic backgrounds display lower levels of moderate to vigorous physical activity compared to those from higher socioeconomic backgrounds [19]. Results from additional studies show that children from low socioeconomic backgrounds may have detrimental levels of health-related fitness because of a lack of access to resources (e.g., before and after school activity programs, play environment/space, etc.) and to parental support needed to sustain healthy physical activity behaviors throughout childhood and into adolescence [20,21,22]. De Greeff et al. [23] confirmed that economically disadvantaged children had relatively low fitness levels compared to children without an economic disadvantage. Moreover, children from low-income families are at higher risk for experiencing physical and mental health problems within high environmental stress [24,25].

Several U.S. states require an annual state-level mandatory fitness assessment for students in public schools [26,27]. FitnessGram [28] is a widely used assessment instrument to measure student fitness levels through a series of fitness tests. FitnessGram seeks non-competitive fitness assessments using criterion-referenced standards to determine student fitness zones. The benefit of this is that the standards used to determine fitness zones could indicate the risk of chronic diseases later in life. Ideally, the FitnessGram can provide physical education teachers, students, and parents with information on students’ fitness levels, and by extension, be used to build a tailored program of physical activity [29]. However, despite various health risk factors due to low level of fitness among children, little research has focused on the fitness levels of children in the United States, and even less research has been conducted on student fitness in Title I schools [30]. Therefore, the purpose of this study was to investigate the health-related fitness levels of Title I elementary school students.

## 2. Materials and Methods

### 2.1. Participants

A total of 77 elementary students (50.6% female; *M*_age_ = 10.0, *SD* = 0.827) from 13 classes of fourth and fifth grades in two Title I elementary schools in the western United States were recruited using convenience sampling [31]. A majority of students (89.6% and 83%, respectively) in these two elementary schools qualified for free and reduced-price lunch [32]. The free and reduced-price lunch program offers federally reimbursable meals for qualified children; students whose household income is less than 130% of the federal poverty level qualify for free lunch, and students whose household income is between 130% and 185% of the federal poverty level qualify for reduced-price lunch [33]. The ethnic composition of the participants consisted of the following: Hispanic 72.8%, Caucasian 18.2%, Asian 6.5%, African American 1.3%, and Native American 1.3%.

### 2.2. Data Collection Procedures

The University’s Institutional Review Board (IRB) approved the study protocol (1498337-2), and all participants’ parents provided informed consent prior to assessment. Over the course of one semester, a team of trained research associates conducted fitness tests during physical education classes with cooperating physical education teachers’ assistance. The team included three teacher education faculty members, six graduate research assistants, approximately 10 undergraduate students from a large teacher preparation institution, and two in-service elementary physical education teachers from the two local Title I elementary schools. All research assistants involved in data collection were trained by a certified Presidential Youth Fitness Program trainer and passed a written knowledge exam of the FitnessGram testing protocols. Students in each class were divided into several small groups (four to six students for each group) and rotated through fitness testing stations administered by research team members. Research team members demonstrated and explained each test, and then students practiced prior to their actual assessment trial.

### 2.3. Instruments

FitnessGram [28] was utilized to measure health-related fitness levels. FitnessGram is one of the most widely used fitness testing systems and is currently used in more than 67,000 schools in the United States [34]. As an integrated fitness and activity assessment program, FitnessGram includes field tests of health-related fitness such as students’ aerobic capacity (i.e., cardiorespiratory endurance), body composition, flexibility, muscular strength, and muscular endurance. In order to allow teachers to provide students with more personalized feedback, FitnessGram also offers its own evaluation criteria, called FitnessGram Healthy Fitness Zone Performance Standards. Numerous studies have determined FitnessGram’s validity and reliability and it is endorsed by the American College of Sports Medicine (ACSM) [35,36,37,38,39]. According to the latest edition of the FitnessGram Administration Manual [28], various test procedures can be designed based upon the primary objective of the program, such as self-testing, individualized testing, and institutional testing. Therefore, school health professionals, including physical education teachers, are recommended to plan for FitnessGram assessments along with their own goal of the test and schools’ environment, including facilities, health support policies, and classroom schedules.

Aerobic Capacity. In order to evaluate aerobic capacity, students completed the Progressive Aerobic Cardiovascular Endurance Run (PACER) test of FitnessGram. Students ran back and forth across a 20-m distance in the schools’ gymnasiums, following signals by a beep sound provided by the Cooper Institute [28]. The pace of the beep sound got progressively faster each minute, and students continued running until they failed to reach the line for a second time before the beep sounded. Students completed the tests in small groups while being observed by researchers to increase the reliability of the test result.

Upper Body Strength and Endurance. Students completed a 90-degree push-up test to measure their upper body strength. Students assumed a plank position with a straight back, and lowered their upper bodies to a 90-degree bend in their elbows and returned to the starting position with arms fully extended. A research team member demonstrated the correct process for the 90-degree push-up test to help students better understand and perform the movement. If a student showed a form error (e.g., touched a knee to the floor), one more opportunity was allowed to continue the test with the correction. After a student performed a second error or chose to stop, the test was concluded. Research team members counted the number of push-ups and checked for the correct form for each student.

Abdominal Strength and Endurance. A curl-up test was utilized to measure students’ abdominal strength and endurance. As a ready position, each student laid on a mat with the back of their head touching the ground, arms outstretched at their sides, and feet flat with knees up. In order for students to demonstrate a successful curl-up, a student curled up slowly by sliding their fingers across the measuring strip to reach the other side of the strip and then curl back down until their head touched the mat. The distance students had to slide their hands varied by age, with nine-year-old students sliding their hands at a 3-inch distance and 10-year-olds (and older students) sliding their hands 5 inches. When performing the curl-up, students were to keep their heels in contact with the mat. Students performed the curl-up as much as they could following the official curl-up cadence provided by the Cooper Institute [28]. Similar to the 90-degree push-up test, if a student showed a form error (e.g., heels lifted up off the mat), one more opportunity to make the correction was permitted. After a student performed a second error or chose to stop, the test concluded. The number of student curl-ups was counted by a research team member, and a demonstration was also conducted by a research team member before the test.

Flexibility. Students also completed a back-saver sit-and-reach test to assess flexibility. Students removed their shoes and placed their right-foot against the measurement box with the leg fully extended and their left-foot flat on the floor with the knee pointing up. Then, students placed both hands on top of each other with palms facing down and reached forward as far as possible three times, holding their outstretched hands the third time as far as they could against the ruler on the measurement box. After completing the back-saver sit-and-reach test with the right leg, the left side was assessed using the same process. Before the assessment, a research team member demonstrated the procedure.

### 2.4. Data Analysis

Descriptive statistics, including frequency distributions, means, and standard deviations, were calculated through SPSS v25.0 [40] to analyze data and identify the number of students in the Healthy Fitness Zone (HFZ) based upon the FitnessGram Healthy Fitness Zone Performance Standards [28].

FitnessGram Healthy Fitness Zone Performance Standards. FitnessGram classifies fitness levels using age- and sex-specific criterion-referenced zones, consisting of the HFZ, the needs improvement (NI) zone, and the needs improvement–health risk (NI–HR) zone (Table 1) [28].

Aerobic Capacity. FitnessGram uses a strategy that converts laps on the PACER test to maximal oxygen uptake (shortly VO2max) to evaluate aerobic capacity in children [28], and it has been validated with elementary-aged children [41]. For this study, laps on the PACER test were converted to VO2max (mL/kg/min) by using Linear Model 2 [42], presented below:

VO2max = 45.619 + (PACER × 0.353) − (Age × 1.121)

For example, a score of 25 laps on the PACER test for a 10-year-old child would be converted to 43.234 of VO2max, and this result is then recorded in the appropriate zone following the FitnessGram Healthy Fitness Zone Performance Standards.

Muscular Strength and Endurance (Upper Body and Abdomen). Assessments for muscular strength and endurance (90-degree push-up and curl-up) did not require conversion. Following Table 1 (FitnessGram Healthy Fitness Zone Performance Standards: Age 9 to 11), raw scores for upper body strength and endurance and abdominal strength and endurance were used to determine placement in the appropriate zone.

Flexibility. As the back-saver sit-and-reach-test was completed with two different sides of the leg, a mean score was calculated. Each student’s score was recorded in the appropriate zone based upon the result of the test.

## 3. Results

**Figure 1:**
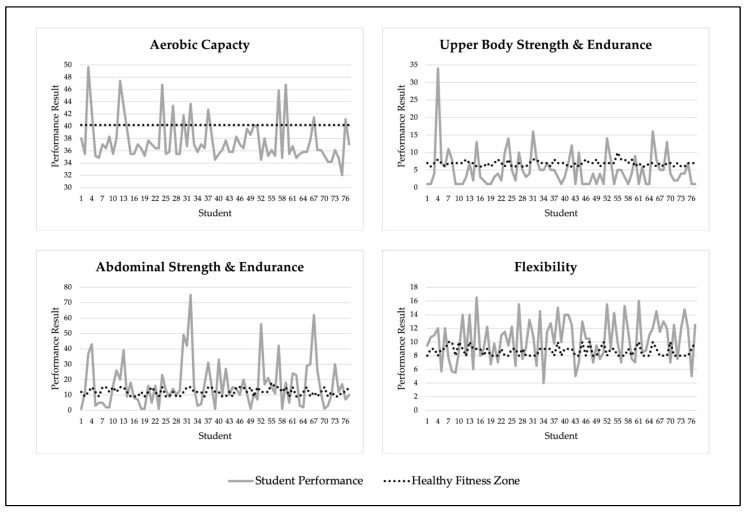
Results for Student Performance.

Table 2 presents means and standard deviations for the results of student performance for all four assessments, and Figure 1 includes four different graphs that show student performance and HFZ performance standards for the four assessments. While PACER test’s HFZ performance standards for all students are the same regardless of gender and age, every student has different standards for the 90-degree push-up, curl-up, and back-saver sit-and-reach tests, in accordance with their gender and age (e.g., HFZ for 9-year-old females for the back-saver sit-and-reach test is ≥9, but for the male is ≥8).

Only 16.9% of students achieved the HFZ for aerobic capacity (7.7% of female students and 26.3% of male students), with 64.9% and 18.2% falling in the NI–HR and NI categories, respectively. Less than a third of students (31.2%) achieved the HFZ for upper body strength and endurance (33.3% of female students and 28.9% of male students) and just over half of students (55.8%) achieved the HFZ for abdominal strength and endurance (59% of female students and 52.6% of male students). More than two-thirds of students (68.8%) achieved the HFZ for flexibility (64.1% of female students and 73.7% of male students).

## 4. Discussion

A large number of studies have examined the prevalence of children living in low-income families and their risk factors (e.g., obesity, bad eating habit, depression, etc.), and the importance of health-related fitness among children was significantly highlighted [5,20,43,44]. Therefore, this study aimed to investigate the health-related fitness levels of Title I elementary school students by utilizing four assessments of FitnessGram [28].

Student assessment results indicated aerobic capacity as the component of greatest concern, with less than 17% of students achieving the HFZ. This is consistent with Burns et al. [45], who found children in low-income schools performed poorly on assessments of aerobic capacity, leading to increased risk of cardiometabolic diseases. Similarly, Bai and colleagues [46] found that HFZ achievement for aerobic capacity of FitnessGram assessment is positively associated with students’ minority rate and low socioeconomic status. Beyond the important connections between aerobic capacity and physical health, high cardiorespiratory fitness is associated with stronger cognitive performance in children [47]. Given this, it is probable that students with lower VO2max may also demonstrate challenges in academic learning.

Only 31.2% of students achieved the HFZ for upper body strength and endurance. In contrast, Bai and colleagues [30] found a HFZ achievement rate of approximately 70% for boys and 58% for girls among a large sample of fourth and fifth graders from 725 schools across the United States. Benson and colleagues [48] offered that children’s muscular strength is associated with insulin sensitivity and suggested that increased muscular strength with specific interventions in children (e.g., strength training) could be beneficial for metabolic fitness. According to the Centers for Disease Control and Prevention (CDC) [49], age appropriate muscle strengthening activities for children include rope or tree climbing, resistance exercises using body weight, climbing on playground equipment, and some forms of yoga. Findings from these studies indicate that additional attention to upper body strength and endurance is warranted in children attending Title I schools.

Just over half of the students achieved the HFZ for abdominal strength and endurance. Recent studies have shown that the prevalence of abdominal obesity among children and adolescents has significantly increased in the United States due to several reasons, especially insufficient exercise and bad eating habits [50,51,52]. This body of research indicates that students attending Title I schools have access to fewer physical activity opportunities, practice unhealthy dietary habits, and may develop abdominal obesity, which accounts for the weak performance of abdomen assessment [19,20]. Burns and Brusseau [53] found that Hispanic children from low-income schools showed weak performance of curl-up assessments, and that is significantly related to cardiometabolic health risk. Beyond school itself, children from low-income environments tend to have insufficient access to physical activity opportunities, including resistance-training activities to improve muscular strength and endurance [5].

Students performed best with respect to flexibility as 68.8% of students achieved the HFZ. It may indicate that the ages of participants for this study are in the phase that has better flexibility, with more soft bones and more flexible tendons and ligaments [54]. Bai et al. [30] also showed similar findings of children’s flexibility with approximately 67% and 72% of boys and girls meeting HFZ criterion, respectively. However, it is difficult to see that the 68.8% is objectively high enough as FitnessGram HFZ criteria have developed according to national databases from long-term field evaluations [39]. Miyamoto et al. [55] highlighted that sit-and-reach tests depend on arm and leg length. For example, children with long arms and short legs or with either one of two features would get better test results in spite of unacceptable hamstring muscle flexibility. With this fact, it is unclear whether or not low-income is directly related to flexibility. However, one additional element to be considered as a potential factor would be ethnic background since the majority of participants in this study were Hispanic (72.8%).

### 4.1. Implications for Practice

Findings from the present study indicate that students from two Title I elementary schools in the western United States did not achieve sufficient levels of health-related fitness across four component areas. Knowing that one of the purposes of quality physical education is to equip students with the “knowledge and skills to achieve and maintain a health-enhancing level of physical activity and fitness” [56], it is important to consider how these findings could inform professional practice. Dauenhauer [57] has proposed a method for tiered intervention where physical educators and other school health professionals use physical activity and fitness data to identify students who are at an elevated health risk. Based on this, interventions can then be implemented to address current and future health concerns in a systematic and preventive manner. The tiered intervention model demonstrated initial efficacy in a low-income elementary school setting, with particularly strong improvements observed in physical activity and aerobic capacity [58]. The model has since been expanded to address both lower- and higher-performing student needs [59] and to align with a broader conceptualization of physical literacy [60]. Tiered interventions such as these can be implemented in low-income schools to promote healthy levels of physical fitness and enhanced physical literacy.

The findings presented here underscore the importance of a focus on health-related fitness for physical educators teaching in Title I schools. More specifically, physical educators can better serve these students by including more activities and lessons focused on aerobic capacity, as this was the component in which students demonstrated the most concerning results. In addition, after-school and at-home programs to encourage moderate-to-vigorous physical activity (MVPA) and provide opportunities for physical activity engagement could also support improved aerobic capacity in students attending Title I schools. Finally, classroom teachers could include small bouts of upper body strength and endurance (e.g., push-ups, modified push-ups, or burpees) easily at various points throughout the school day.

### 4.2. Limitations and Future Research Lines

There are several limitations to this present study. First, in terms of the sample, the number of participants who were purposefully recruited using convenience sampling were only from two Title I schools in the western United States; this led to a lack of diversity. The majority of participants were Hispanic children, and this, along with the narrow age range of students primarily between the ages of 9–11 years, may limit the generalizability of the study to other populations of children with different ethnicities and ages (e.g., adolescents). Additionally, further exploration is needed to examine the fitness discrepancies in several potential contextual factors (e.g., racial/ethnic factors, geographic influence, school and/or neighborhood environments, the quality, and amount of physical education, etc.) with a larger sample. Second, body composition, as one of the health-related fitness components, was not included as a part of the student fitness assessment for this study. FitnessGram uses Body Mass Index (BMI) to measure child body composition, but various studies have pointed out that BMI can lead to misinformation and confusion about the degree of individual obesity [61,62]. According to research, BMI does not account for bone density, muscle mass, and racial differences, so it does not accurately present overall body composition. Moreover, BMI measurement in school settings can be a sensitive topic, particularly when personal privacy is difficult to maintain [63]. Therefore, the research team decided to forego the collection of these data. Although there was a reason for not measuring BMI for this study, body composition is still one of the health-related fitness components, so future studies should consider including a measure of body composition to complete assessing all five health-related fitness components, as it may lead to better presentation of the overall health-related fitness levels of participants.

## 5. Conclusions

Despite the known benefits of optimal health-related fitness levels in youth, a number of studies have highlighted that children living in low-income households have less opportunity for physical activity participation. The results of this study offer insights into the health-related fitness levels of a unique population, students attending Title I schools. School health professionals, including physical education teachers, need to be aware of existing disparities and make efforts to systematically intervene. Further, physical educators should ensure that they are considering appropriate practices for fitness education [63], and when working with students—especially those from Title I schools—that they are providing equitable and inclusive opportunities for fitness development.

## Figures and Tables

**Table 1:** FitnessGram Healthy Fitness Zone Performance Standards: Age 9 to 11 [28].

Age	Aerobic Capacity			Upper Body Strength and Endurance	Abdominal Strength and Endurance	Flexibility
	PACER (VO2max—mL/kg/min)			90-Degree Push-Up (Number of Repetitions)	Curl-Up (Number of Repetitions)	Back-Saver Sit-and-Reach (inch)
	NI–HR	NI	HFZ	HFZ	HFZ	HFZ
9	≤37.3	37.4–40.1	≥40.2	F: ≥6 M: ≥6	F: ≥9 M: ≥9	F: ≥9 M: ≥8
10	≤37.3	37.4–40.1	≥40.2	F: ≥7 M: ≥7	F: ≥12 M: ≥12	F: ≥9 M: ≥8
11	≤37.3	37.4–40.1	≥40.2	F: ≥7 M: ≥8	F: ≥15 M: ≥15	F: ≥10 M: ≥8

Note: F = female; M = male; NI–HR = needs improvement–health risk; NI = needs improvement; HFZ = healthy fitness zone.

**Table 2:** Results for Student Performance.

Biological Sex	Aerobic Capacity		Upper Body Strength and Endurance		Abdominal Strength and Endurance		Flexibility	
	PACER (VO2max—mL/kg/min)		90-Degree Push-Up (Number of Repetitions)		Curl-Up (Number of Repetitions)		Back-Saver Sit-and-Reach (inches)	
	M	SD	M	SD	M	SD	M	SD
Female (*n* = 39)	37.20	2.77	4.69	3.84	14.10	10.29	10.60	3.49
Male (*n* = 38)	38.18	4.05	5.74	6.26	19.26	18.65	10.04	2.78
Overall (*N* = 77)	37.67	3.47	5.21	5.17	16.65	15.13	10.32	3.15

Note: M = mean; SD = standard deviation.

## References

[1] Morrow J.R., Tucker J.S., Jackson A.W., Martin S.B., Greenleaf C.A., Petrie T.A. (2013). Meeting physical activity guidelines and health-related fitness in youth. Am. J. Prev. Med..

[2] Ortega F.B., Ruiz J.R., Castillo M.J., Sjöström M. (2008). Physical fitness in childhood and adolescence: A powerful marker of health. Int. J. Obes..

[3] Ruiz J.R., Castro-Piñero J., Artero E.G., Ortega F.B., Sjöström M., Suni J., Castillo M.J. (2009). Predictive validity of health-related fitness in youth: A systematic review. Br. J. Sports Med..

[4] Marshall S.J., Sarkin J.A., Sallis J.F., McKenzie T.L. (1998). Tracking of Health-Related Fitness Components in Youth Ages 9 to 12. Med. Sci. Sports Exerc..

[5] Brusseau T.A., Hannon J., Burns R. (2016). The effect of a comprehensive school physical activity program on physical activity and health-related fitness in children from low-income families. J. Phys. Act. Health.

[6] Ganley K.J., Paterno M.V., Miles C., Stout J., Brawner L., Girolami G., Warren M. (2011). Health-related fitness in children and adolescents. Pediatr. Phys. Ther..

[7] Centeio E.E., Somers C.L., Moore W.G., Garn A., Kulik N., Martin J., Shen B., McCaughtry N. (2020). Considering physical well-being, self-perceptions, and support variables in understanding youth academic achievement. J. Early Adolesc..

[8] Srikanth S., Petrie T.A., Greenleaf C., Martin S.B. (2015). The relationship of physical fitness, self-beliefs, and social support to the academic performance of middle school boys and girls. J. Early Adolesc..

[9] Bull F.C., Al-Ansari S.S., Biddle S., Borodulin K., Buman M.P., Cardon G., Carty C., Chaput J.-P., Chastin S., Chou R. (2020). World Health Organization 2020 Guidelines on Physical Activity and Sedentary Behaviour. Br. J. Sports Med..

[10] US Department of Health and Human Services (2018). Physical Activity Guidelines for Americans.

[11] Katzmarzyk P.T., Denstel K.D., Beals K., Carlson J., Crouter S.E., McKenzie T.L., Pate R.R., Sisson S.B., Staiano A.E., Stanish H. (2018). Results from the United States 2018 Report Card on Physical Activity for Children and Youth. J. Phys. Act. Health.

[12] Poitras V.J., Gray C.E., Borghese M.M., Carson V., Chaput J.-P., Janssen I., Katzmarzyk P.T., Pate R.R., Connor Gorber S., Kho M.E. (2016). Systematic review of the relationships between objectively measured physical activity and health indicators in school-aged children and youth. Appl. Physiol. Nutr. Metab..

[13] Armstrong S., Wong C.A., Perrin E., Page S., Sibley L., Skinner A. (2018). Association of physical activity with income, race/ethnicity, and sex among adolescents and young adults in the United States: Findings from the National Health and Nutrition Examination Survey, 2007-2016. JAMA Pediatr..

[14] Brownson R.C., Boehmer T.K., Luke D.A. (2005). Declining rates of physical activity in the United States: What are the contributors?. Annu. Rev. Public Health..

[15] Fakhouri T.H., Hughes J.P., Brody D.J., Kit B.K., Ogden C.L. (2013). Physical activity and screen-time viewing among elementary school–aged children in the United States from 2009 to 2010. JAMA Pediatr..

[16] Katz D.L., Cushman D., Reynolds J., Njike V., Treu J.A., Katz C., Walker J., Smith E. (2010). Peer reviewed: Putting physical activity where it fits in the school day: Preliminary results of the ABC (Activity Bursts in the Classroom) for fitness program. Prev. Chronic Dis..

[17] McLaughlin M.W. (1975). Evaluation and Reform: Elementary and Secondary Education Act of 1965, Title 1.

[18] National Center for Education Statistics (2015). Study of the Title I, Part A Grant Program Mathematical Formulas.

[19] Borraccino A., Lemma P., Iannotti R.J., Zambon A., Dalmasso P., Lazzeri G., Giacchi M., Cavallo F. (2009). Socio-economic effects on meeting PA guidelines: Comparisons among 32 countries. Med. Sci. Sports Exerc..

[20] Fu Y., Brusseau T.A., Hannon J.C., Burns R.D. (2017). Effect of a 12-week summer break on school day physical activity and health-related fitness in low-income children from CSPAP schools. J. Environ. Public Health..

[21] Janssen I., LeBlanc A.G. (2010). Systematic review of the health benefits of physical activity and fitness in school-aged children and youth. Int. J. Behav. Nutr. Phys. Act..

[22] Lampard A.M., Jurkowski J.M., Lawson H.A., Davison K.K. (2013). Family ecological predictors of physical activity parenting in low-income families. Behav. Med..

[23] De Greeff J.W., Hartman E., Mullender-Wijnsma M.J., Bosker R.J., Doolaard S., Visscher C. (2014). Physical fitness and academic performance in primary school children with and without a social disadvantage. Health Educ. Res..

[24] Hodgkinson S., Godoy L., Beers L.S., Lewin A. (2017). Improving mental health access for low-income children and families in the primary care setting. Pediatrics.

[25] Odgers C.L., Adler N.E. (2018). Challenges for low-income children in an era of increasing income inequality. Child Dev..

[26] Plowman S.A., Sterling C.L., Corbin C.B., Meredith M.D., Welk G.J., Morrow J.R. (2006). The history of FITNESSGRAM^®^. J. Phys. Act. Health.

[27] Pluim C., Gard M. (2018). Physical education’s grand convergence: Fitnessgram^®^, big-data and the digital commerce of children’s health. Crit. Stud. Educ..

[28] Cooper Institute (2017). Fitnessgram Administration Manual.

[29] Kohl H.W., Cook H.D. (2013). Educating the Student Body: Taking Physical Activity and Physical Education to School.

[30] Bai Y., Saint-Maurice P.F., Welk G.J., Allums-Featherston K., Candelaria N., Anderson K. (2015). Prevalence of youth fitness in the United States: Baseline results from the NFL PLAY 60 FITNESSGRAM partnership project. J. Pediatr..

[31] Hall S., Getchell N. (2014). Research Methods in Kinesiology, and the Health Sciences.

[32] News Release—Colorado announces 2017–2018 Free and Reduced-Price Meal Income Guidelines. https://www.cde.state.co.us/communications/20170705freeandreducedguidelines.

[33] Domina T., Pharris-Ciurej N., Penner A.M., Penner E.K., Brummet Q., Porter S.R., Sanabria T. (2018). Is free and reduced-price lunch a valid measure of educational disadvantage?. Educ. Res..

[34] FITNESSGRAM Selected as Statewide Fitness Tool. https://www.cooperinstitute.org/pub/news.cfm?id=24.

[35] Baumgartner T.A., Oh S., Chung H., Hales D. (2002). Objectivity, reliability, and validity for a revised push-up test protocol. Meas. Phys. Educ. Exerc. Sci..

[36] Hashim A., Ariffin A., Hashim A.T., Yusof A.B. (2018). Reliability and Validity of the 90º Push-Ups Test Protocol. Int. J. Sci. Res..

[37] Morrow J.R., Martin S.B., Jackson A.W. (2010). Reliability and validity of the FITNESSGRAM^®^: Quality of teacher-collected health-related fitness surveillance data. Res. Q. Exerc. Sport..

[38] Sherman T., Barfield J.P. (2006). Equivalence reliability among the FITNESSGRAM^®^ upper-body tests of muscular strength and endurance. Meas. Phys. Educ. Exerc. Sci..

[39] Welk G.J., Going S.B., Morrow J.R., Meredith M.D. (2011). Development of new criterion-referenced fitness standards in the FITNESSGRAM^®^ program: Rationale and conceptual overview. Am. J. Prev. Med..

[40] IBM Corp (2017). IBM SPSS Statistics for Windows, Version 25.0.

[41] Cureton K.J., Mahar M.T. (2014). Critical measurement issues/challenges in assessing aerobic capacity in youth. Res. Q. Exerc. Sport..

[42] Mahar M.T., Guerieri A.M., Hanna M.S., Kemble C.D. (2011). Estimation of aerobic fitness from 20-m multistage shuttle run test performance. Am. J. Prev. Med..

[43] Manson J., Rotondi M., Jamnik V., Ardern C., Tamim H. (2013). Effect of tai chi on musculoskeletal health-related fitness and self-reported physical health changes in low income, multiple ethnicity mid to older adults. BMC Geriatr..

[44] Veugelers P.J., Fitzgerald A.L. (2005). Prevalence of and risk factors for childhood overweight and obesity. CMAJ.

[45] Burns R.D., Brusseau T.A., Fang Y., Fu Y., Hannon J.C. (2016). Waist-to-Height ratio, aerobic fitness, and cardiometabolic risk in hispanic children from low-income US Schools. Pediatr. Exerc. Sci..

[46] Bai Y., Saint-Maurice P.F., Welk G.J., Allums-Featherston K., Candelaria N. (2016). Explaining disparities in youth aerobic fitness and body mass index: Relative impact of socioeconomic and minority status. J. Sch. Health.

[47] Chaddock L., Hillman C.H., Pontifex M.B., Johnson C.R., Raine L.B., Kramer A.F. (2012). Childhood aerobic fitness predicts cognitive performance one year later. J. Sports Sci..

[48] Benson A.C., Torode M.E., Singh M.A.F. (2006). Muscular strength and cardiorespiratory fitness is associated with higher insulin sensitivity in children and adolescents. Int. J. Pediatr. Obes..

[49] CDC Aerobic, Muscle- and Bone-Strengthening: What Counts for School-Aged Children and Adolescents?. https://www.cdc.gov/physicalactivity/basics/children/what_counts.htm.

[50] Gaston S.A., Tulve N.S., Ferguson T.F. (2019). Abdominal obesity, metabolic dysfunction, and metabolic syndrome in US adolescents: National Health and Nutrition Examination Survey 2011–2016. Ann. Epidemiol..

[51] Geiger S.D., Yao P., Vaughn M.G., Qian Z. (2021). PFAS exposure and overweight/obesity among children in a nationally representative sample. Chemosphere.

[52] Xi B., Mi J., Zhao M., Zhang T., Jia C., Li J., Zeng T., Steffen L.M. (2014). Trends in abdominal obesity among US children and adolescents. Pediatrics.

[53] Burns R.D., Brusseau T.A. (2017). Muscular strength and endurance and cardio-metabolic health in disadvantaged Hispanic children from the US. Prev. Med..

[54] Brown J., Fishman L.E. (2017). Kid care on the slopes. Contemp. Pediatr..

[55] Miyamoto N., Hirata K., Kimura N., Miyamoto-Mikami E. (2018). Contributions of hamstring stiffness to straight-leg-raise and sit-and-reach test scores. Int. J. Sports Med..

[56] SHAPE America—Society of Health and Physical Educators (2015). The Essential Components of Physical Education.

[57] Dauenhauer B.D. (2012). Applying response to intervention in physical education. Strategies.

[58] Dauenhauer B., Keating X., Lambdin D. (2016). Effects of a three-tiered intervention model on physical activity and fitness levels of elementary school children. J. Prim. Prev..

[59] Dauenhauer B., Keating X., Lambdin D., Knipe R.K. (2017). A conceptual framework for tiered intervention in physical education. J. Phys. Educ. Recreat. Dance.

[60] Ha T., Dauenhauer B. A physical literacy index: Identifying students for intervention through standards-based assessment. J. Phys. Educ. Recreat. Dance.

[61] Blackburn H., Jacobs D. (2014). Commentary: Origins and evolution of body mass index (BMI): Continuing saga. Int. J. Epidemiol..

[62] De Lorenzo A., Romano L., Di Renzo L., Gualtieri P., Salimei C., Carrano E., Rampello T., de Miranda R.C. (2019). Triponderal mass index rather than body mass index: An indicator of high adiposity in Italian children and adolescents. Nutrition.

[63] SHAPE America—Society of Health and Physical Educators (2017). Appropriate and Inappropriate Practices Related to Fitness Testing.

